# Ultrasound-guided lateral branch radiofrequency neurotomy for sacroiliac joint pain after lumbosacral spinal fusion surgery

**DOI:** 10.1038/s41598-023-33960-z

**Published:** 2023-04-24

**Authors:** Viet-Thang Le, Anh Minh Nguyen, Phuoc Trong Do

**Affiliations:** 1 Pain Management Unit, Department of Neurosurgery, University Medical Center, Ho Chi Minh City, 700000 Vietnam; 2 University of Medicine and Pharmacy at Ho Chi Minh City, Ho Chi Minh City, 700000 Vietnam

**Keywords:** Neuroscience, Diseases

## Abstract

Our study evaluated the clinical feasibility of ultrasound-guided lateral branch radiofrequency neurotomy for sacroiliac joint (SIJ) pain after lumbosacral spinal fusion surgery (LSFS). This prospective study included a total of 46 patients who were diagnosed with SIJ pain after LSFS, did not respond to conservative treatment and therefore underwent ultrasound-guided SIJ radiofrequency neurotomy (RFN) from January 2019 to January 2022. These patients were followed up for twelve months after the procedure. Patients were assessed with the Numeric Rating Scale (NRS) and the Oswestry Disability Index (ODI) preprocedural and postprocedural for 1 month, 6 months and 12 months follow-ups. There was a significant improvement in postprocedural NRS and ODI scores (*p* < 0.001). Thirty-eight patients (82.6%) had a satisfactory response and good global perceived effect (GPE) after twelve months. No significant complications were observed during the 12-month follow-up. The ultrasound-guided radiofrequency device designed as a safe, easily applied and encouraging method could avoid revision surgery. It is a promising technique and has shown good results in providing intermediate pain relief. In addition to the limited series reported in the literature, future studies will add meaning to this topic by including it in routine practice.

## Introduction

Spinal fusion surgery performed in carefully selected patients with an adult spinal deformity has improved clinical outcomes when attention is given to restoring spinopelvic sagittal alignment parameters. Despite this, the frequency with which surgery had failed to relieve symptoms remains as high as 20–40%^1^. Instead of categorically labeling this group of patients with “failed back surgery syndrome—FBSS”, it is essential to identify possible causes for their ongoing or new onset symptoms. A potential reason might be pain originating from the SIJ. The prevalence of SIJ pain due to FBSS is estimated to be 29%^2^. SIJ is the most likely source of low back pain, particularly in patients undergoing lumbar and sacral fusion surgery.

The SIJ creates the lowest segment of the spinal axis and is the largest axial joint in the body^3^. Lumbosarcral spinal fusion surgery (LSFS) results in increased stress on the SIJ. The mechanical response seen in SIJ after spinal fusion is similar to that occurring in the mobile segments adjacent to the fused spine, with increased mobility and an increase in surface stress, and articular surfaces were confirmed by a biomechanical study. The frequency with which SIJ pain contributes to persistent low back pain after LSFS has been reported to range from 32 to 42%^2,4,5^. There has been an increase in the number of lumbar and sacral fusion surgeries over the past 10 years^6^. In a multicenter study, it was reported that such surgical procedures yielded better improvement than conservative treatment. Following fusion surgery, some patients may have recurrent and new low back pain that is different from that seen before surgery. Almost all prior injection techniques under sonography required firm solutions by fluoroscopy or computer tomography (CT) images, and our study used ultrasound guidance to perform RFN for SIJ pain after LSFS. Ultrasound guidance is increasingly being explored for a variety of interventional spine procedures. Ultrasound (US) imaging has significant advantages, as it is easily accessible and does not use ionizing radiation, portability, accessible to US equipment, and the reduced cost of US equipment. This study aimed to evaluate the preliminary clinical results of our proposed US-guided SIJ RFN technique. The outcome of this study was the changes in pain intensity and function according to the US-guided SIJ RFN. We hypothesized that the US-guided SIJ RFN would significantly reduce SIJ pain after lumbar fusion and improve function and quality of life.

## Results

### Patient characteristics

There were forty-six eligible patients. The mean age was 51.7 ± 15.3 years old, and the majority were male patients (32 males, 14 females). Before the procedure, pain was assessed by NRS and ODI (30.4% of patients with cripples). The characteristics of the patients are presented in Table 1.

**Table 1:** Patient Characteristics and Baseline Information on Admission.

Characteristic	N = 46
Age (mean ± SD, year)	51.7 ± 15.3 (range, 38–79)
Sex, M/F	32/14
Fusion to sacrum	20 (43.5%)
NRS (mean ± SD)	7.33 ± 1.05
NRS (fusion to L5)	
Baseline	6.65 ± 0.99
12 months	3.30 ± 0.58
NRS (fusion to S1)	
Baseline	7.85 ± 0.78
12 months	3.60 ± 0.82
ODI	
21–40%	26.1%
41–60%	43.5%
61–80%	30.4%
81–100%	0
Pain duration (mean ± SD, month)	17.9 ± 2.5 (range, 6–54)
Number of fused segments	
One	12 (26.1%)
Two	15 (32.6%)
Three	11 (23.9%)
≥ 4	8 (17.4%)
Unilateral/bil ateral pain	18/28
Good GPE (12 months)	38 (82.6%)

### Effectiveness of radiofrequency neurotomy with longitudinal data

**Figure 1:**
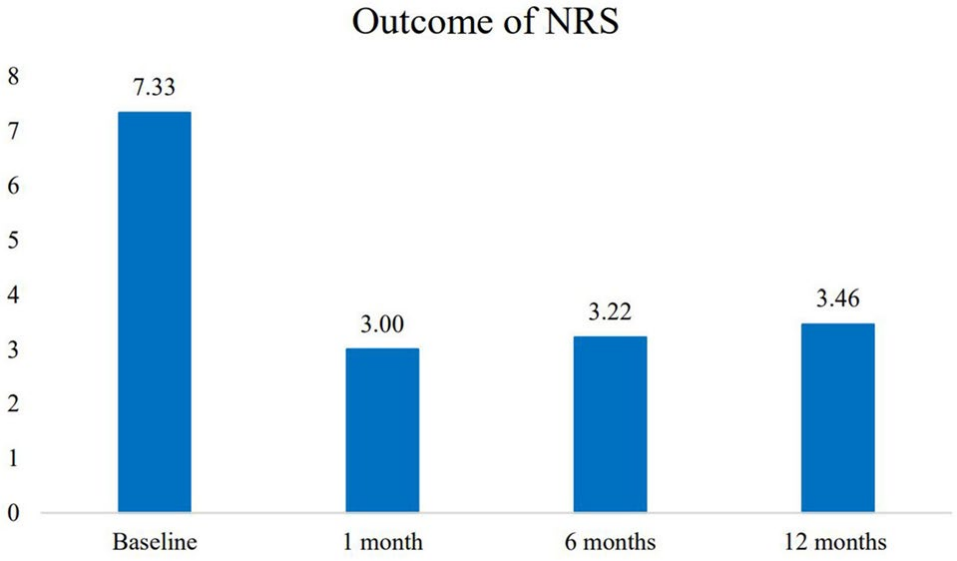
The outcome measured by NRS (Software: Adobe Inc., 2019. Adobe Photoshop, Available at: https://www.adobe.com/products/photoshop.html).

The NRS score one week after the procedure was significantly lower than that before the injection at admission (*p* < 0.001). The NRS at the one-month, six-month and twelve-month reexaminations showed a reduction versus the pre intervention NRS (Fig. 1).

The terms of the Oswestry Disability Index, the ODI before RFN was 59.1 ± 13.6 (mean, SD). A significant difference in ODI was detected after RFN. The ODI mean (± SD) at 1, 6, and 12 months after the procedure was 23.9 ± 7.6, 24.8 ± 8.5, and 30.0 ± 12.0, respectively. The suffered cripple patients (30.8%) improved function early in the first month of post-procedural, there was no patient have cripple ODI after 12 months of treatment (0%). Patients who received radiofrequency treatment reported significantly lower ODI scores at 1, 6, and 12 months compared to their baseline scores. The change in ODI before and after the intervention at 1, 6, and 12 months is statistically significant (*p* < 0.001). The outcome measured by ODI is presented in Table 2.

**Table 2:** Outcome measured by the oswestry disability index.

Level of disability	Time			
	Baseline N = 46	1-month N = 46	6-months N = 46	12-months N = 46
Minimal (0–20)	0	58.7	71.7	65.2
Moderate (21–40)	26.1	32.6	23.9	26.1
Severe (41–60)	43.5	8.7	4.4	8.7
Cripple (61–80)	30.4	0	0	0
Bed-bound (81–100)	0	0	0	0
*p* value (T-test)		0.001	0.001	0.001

### Safety

The injection procedures were tolerable for all the patients. No patient experienced any complications, such as aggravation of pain, numbness, headache, dizziness, or an allergic reaction. There were no treatment-related complications after 12 months of follow-up.

## Discussion

The most common painful sites for SIJ pain are the hips (94%), followed by the lower lumbar region (72%), lower extremities (50%), groin area (14%), upper lumbar region (6%) and abdomen (2%). Maigne and Planchon have suggested that the only criteria characterizing SIJ pain following lumbar fusion are a distribution pattern of pain different from that present before surgery. Almost all of the patients had pain distributed to the hips, back and side of the thighs^4^. The most challenging aspect of treatment for SIJ pain is the difficulty of the diagnosis because there are no specific examination findings. Slipman et al. found the positive predictive value of three positive responses to provocative tests to be 60%; however, Broadhurst and Bond reported 77–87% sensitivity for three positive responses to provocative tests^7,8^. For the diagnosis of SIJ pain in our patients experiencing pain following fusion surgery, multiple provocative tests of sacral sulcus compression, FABER and Gaenslein tests were used. Of these patients, 18 (39.1%) had unilateral and 28 (60.8%) had bilateral SIJ-originated pain. The use of multiple maneuvers for provoking SIJ pain is a reliable test for the presence of SIJ pain. Thus, three positive SIJ provocation tests are important in terms of diagnostic. However, we found no studies that exclusively evaluated stimulation tests in patients with SIJ pain after sacral surgery^7–10^.

The most commonly reported pain in adults is low back pain. In addition to being an economic burden for society, a growing and expanding series of patients with low back pain have been observed. There has been an increase in lumbar lumbosacral fusion surgery in the last 10 years. Some authors have suggested that the SIJ plays a role in postoperative pain experienced by patients undergoing lumbar fusion surgery^3–6^. One of the three possible causes of SIJ pain is increased mechanical load transfer onto the SIJ after lumbar or lumbosacral fusion. Ha et al. found a higher incidence of SIJ degeneration in patients with fusion to S1 than in those with fusion to L5^11^. All the above mentioned studies have reported similar results to the results of our research.

The results of this study are a group of patients with fusion ending at S1 had more severe pain than the L5 group of patients. The mean NRS score was 7.85 in the S1 group and 6.65 in the L5 group in our study. The S1 group of patients had more severe pain than the L5 group. Our study indicated that the mean NRS score significantly improved in the L5 fusion group from 6.65 at the time of onset to 3.30 at the time of final follow-up; however, in the S1 fusion group, it improved from 7.85 at the time of onset to 3.60 at the time of final follow-up. In this study, patients who received an S1 sacral fusion had a lower response than the L5 and above vertebrae stiffening group, but this difference was not statistically significant. Ha et al. also found no significant difference in the decrease after the procedure in VAS scores between the 2 groups' fusion to L5 and S1 (*p* = 0.145)^11^.

In Vietnam, this prospective observational study is the first to evaluate the use of a US-only-guided technique for SIJ RFN. The findings of this study suggest that the US-guided posterior sacral network (PSN) lateral crest RFN technique may reduce pain intensity, improve function and improve quality of life for several months post-RFN in those with SIJ pain. Our findings showed a reduction in NRS from 7.33 before the procedure to 3.00 after one month and to 3.46 after a twelve-month follow-up. This study found a significant decrease in SI joint-related pain after radiofrequency neurotomy under ultrasound into the SI joint in patients who still had pain after lumbosacral fusion. This diagnostic strategy confirmed the clinical diagnosis of SIJ syndrome after lumbosacral fusion in 88.5% (54/61) of patients. Our results in 46 patients demonstrated the short-term radiofrequency denervation efficacy of SIJ pain management. No serious complications or side effects were observed in our patients. Almost all of the patients showed a clinically relevant degree of pain relief, with at least a three-point reduction in NRS for pain, representing a statistically significant reduction in mean pain intensity scores.

We also noted a trend toward better outcomes based on ODI. In terms of ODI, which indicated the level of disability, there were no cripple patients post-procedural after the one-month and twelve-month follow-ups. Most patients had a better quality of life and even returned to work. The reduction of post-procedural analgesic consumption and ODI were reported in Omar’s research, which studied the effect on 32 patients with radicular pain. ODI decreased significantly after intervention up to 12 months compared to the post-procedural value. GPE for patient satisfaction was positive in 82.6% of patients. Evaluation of GPE 12 months after the procedure was considered a positive response if the patient reported a decrease in pain and an improved ability to perform daily activities and if the patient introduced this treatment to other patients.

In recent years, the denervation of sacral lateral branches innervating the SIJ via RF energy has drawn great attention. RF applications for the treatment of chronic pain originating from the SIJ are relatively newer than those used for facet joint syndrome, and there are no large case series in the literature. The mechanism by which RF denervation alleviates pain sensation consists of applying an electrical current generated by radio waves to heat nerve fibers and thus reduce pain signals^12,13^. In 2001, Ferrante et al. reported the first bipolar RF technique employed in treating SI joint disorder by creating a strip lesion of the posterior SI joint with RF needles inserted at < 1 cm intervals. As a result, 36% of the patients experienced 50% pain relief for 6 months^14^. Afterward, conventional unipolar (or monopolar) RF targeting the lateral branches of the primary dorsal rami was adopted in several studies, which resulted in sustained relief for 6 months in over 60% of subjects^15,16^. This study utilized a lateral branch block technique similar to the US technique evaluated by Finlayson et al. This technique involved the infusion of local anesthetic at TST1, TST2 and TST3, while the block technique in this study utilized sequential infusion of local anesthetic along the lateral crest from TST1 to TST3 at 1 cm intervals^17^. The proportion of patients achieving at least a 50% reduction in pain intensity 1–12 months post-RFN was high. In addition, although care was taken by the interventionalists to ensure that the RFN cannulas were placed parallel to each other, parallel placement was challenging to achieve at TST1. The prominence of the PSIS made a purely sagittal, out-of-plane approach to TST1 difficult; therefore, an approach that was between an out-of-plane and in-plane approach was necessary to reach TST1. If the distance between electrodes at the periosteal level was too far apart, an adequate lesion may not have been generated.

In the future, a randomized trial comparing active and placebo US-guided SIJ RFN techniques would confirm the preliminary findings of this study. The use of a standardized dual US-guided block protocol within the randomized trial would be necessary to minimize false positive diagnostic blocks. To improve clinical outcomes, future studies should also evaluate the characteristics of patients who have a high likelihood of achieving significant pain relief. The factors that can optimize lesion generation for SIJ RFN, such as tip spacing, lesion duration and cannula type, should also be evaluated. In conclusion, clinical outcomes following US-guided SIJ RFN are promising. These findings suggest that US could be used to guide SIJ RFN. Further randomized controlled studies are necessary to establish the effectiveness of this technique.

The high success rate in our patient series may be associated with the fact that lumbosacral fusion surgery was included in the present study. We believe that percutaneous interventional RF applications should be used in neurosurgical operations for the diagnosis/treatment of low back pain following fusion surgery. SIJ pain should be considered, particularly in patients with pain radiating to the hips and back of the thigh following fusion surgery with fusion ending at the level of S1 and L5. The ultrasound-guided RF device designed as a safe, easily applied and encouraging method will eliminate unnecessary and accurate surgical interventions and revision surgeries.

There are some limitations in this study. First, there was no definitive control group; neither the patients nor the doctor was blinded. Second, this was not a double-blind, placebo-controlled trial. This design was not chosen because the patient was in severe pain and it was considered unethical to use a placebo. Third, data were affected by lost-contact patients during follow-up. The long-term effects of this procedure, the effectiveness of SIG tests, and the safety of the used RFN require a more significant number of studies in the future. In addition to the limited series reported in the literature, future studies will add meaning to this topic by including it in routine practice.

## Methods

This prospective study was carried out on 46 patients who previously underwent lumbar or lumbosacral fusion surgery and were admitted to our outpatient clinic between January 2019 and January 2022 with postoperative persistent chronic pain (more than 6 months), which is different from preoperative pain.

Patients were included if they met the following criteria:

- Lumbar fusion that involved L5 or S1
- Pain consistent with SIJ origin graded on NRS ≥ 5, unilateral or bilateral pain.
- 50% pain relief after sacral lateral branch block.
- Positive Fortin sign (defined in the original article as the site of maximum pain, designated by one finger within 1 cm inferomedial to the posterosuperior iliac crest).
- No satisfactory improvement of symptoms with conservative treatment: non-steroidal anti-inflammatory drugs, tramadol, gabapentinoids (gabapentin or pregabalin) and physical therapy.

The following exclusion criteria were used:

- Symptomatic lumbar pathology.
- Inflammatory, tumoural, traumatic or infectious disease of the SIJ.
- Hip osteoarthritis or history of hip arthroplasty.
- No disc herniation, instability or pseudoarthrosis.
- Symptoms of severe nerve damage including motor paralysis, muscle atrophy, peripheral neuropathy, arterial disease, malignancy and cauda equina syndrome.
- Allergy to the drugs used in this study.
- Prior epidural injection in the past 3 months: nerve root injection and caudal injection.
- Coagulation disorder.
- Pregnancy.

### Clinical and radiological assessment

In addition to the Fortin test, the following clinical signs were met in our population:

- At least 3 positive physical examination maneuvers: Faber (flexion abduction and external rotation), POSH (posterior shear), REAB (resisted abduction), Gaenslen's test and distraction test.
- Pain at the trochanteric insertion of the gluteus medius.
- Maximum pain in a seated position.

The sacroiliac imaging work-up consisted of the following:

- Radiographs and MRI of the lumbar spine and hips.
- Radiographs centered over the SI joints.
- MRI to look for degenerative or inflammatory changes in the joint.

### SIJ procedure

**Figure 2:**
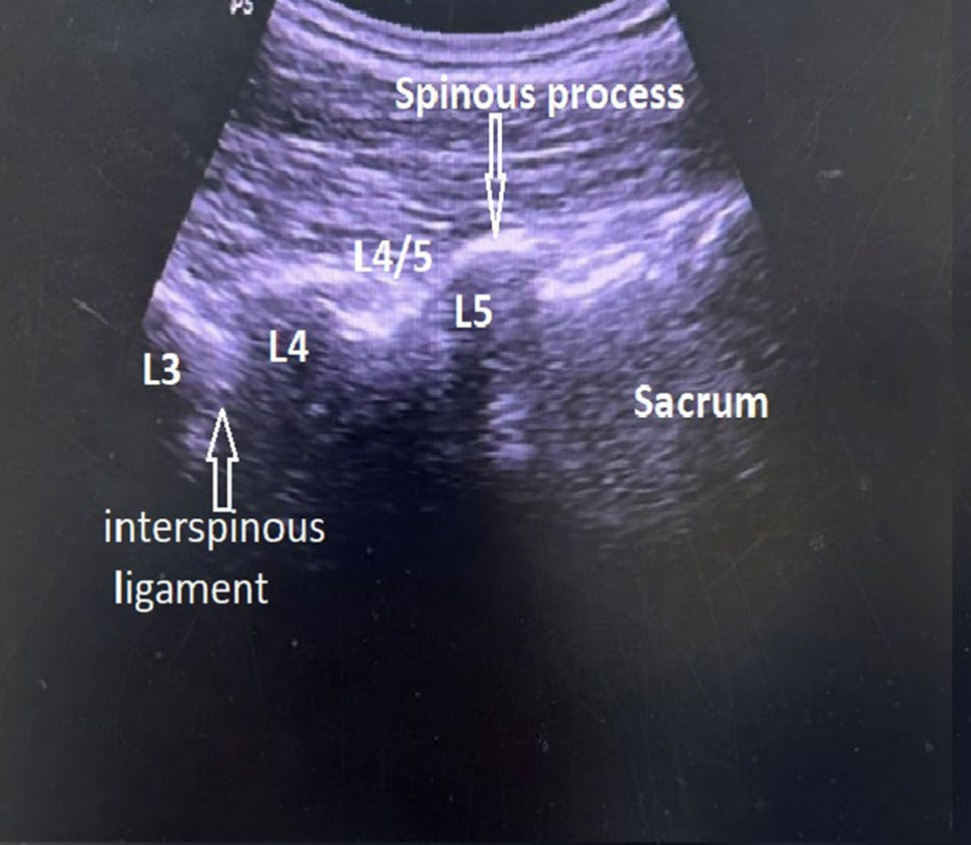
Definition of the sacrum (Software: Adobe Inc., 2019. Adobe Photoshop, Available at: https://www.adobe.com/products/photoshop.html).

**Figure 3:**
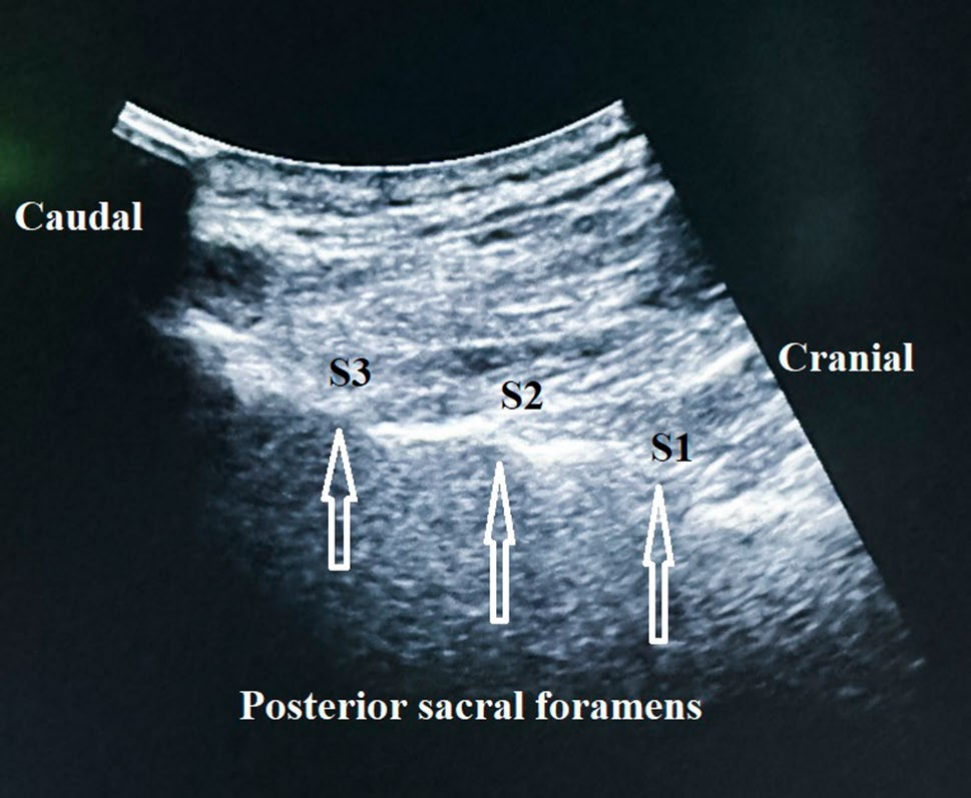
Posterior sacral foramens (PSF) on the sagittal plane (Software: Adobe Inc., 2019. Adobe Photoshop, Available at: https://www.adobe.com/products/photoshop.html).

**Figure 4:**
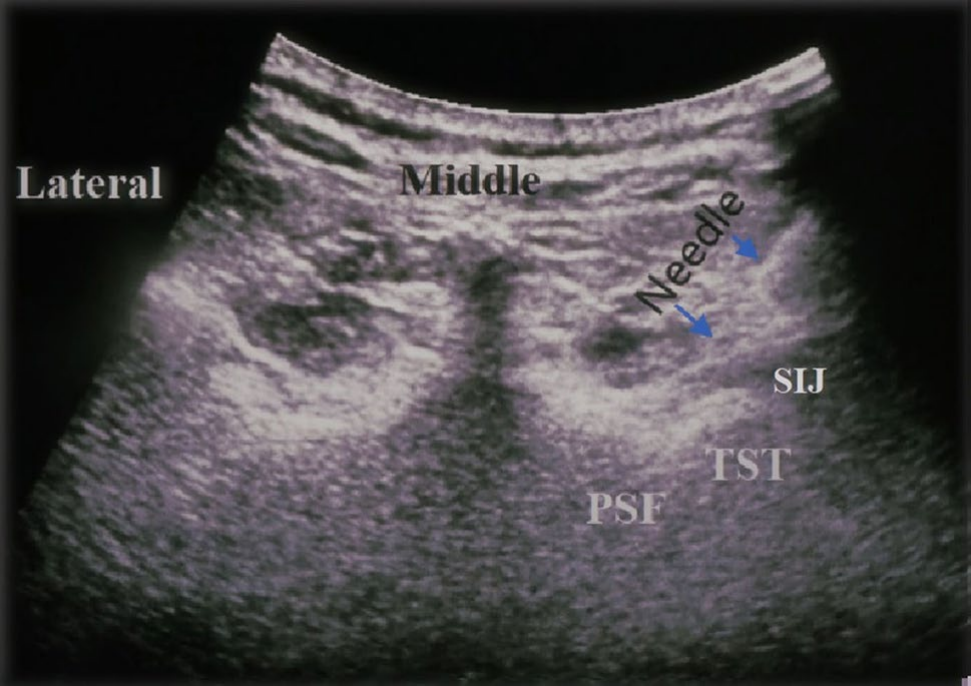
In-plane approach of the lateral branch block technique (Software: Adobe Inc., 2019. Adobe Photoshop, Available at: https://www.adobe.com/products/photoshop.html).

A diagnostic test was performed for patients with LBP after lumbosarcral spinal fusion surgery was resistant to medical treatment and met the criteria through clinical and imaging examination. A standardized US-guided lumbar approach was utilized for pararadicular spinal injections by LOGIQ P5 (device signature P5: 178,256 SU4). A 2–5 MHz convex probe and a prone position were required. Initially, the sacrum was defined in a sagittal US image in the midline plane starting from the spinous processes to the caudal patient's body (Fig. 2). The bony landmarks used to define the lateral sacral crest where the PSN extends were the posterior sacral foramen (PSF) and the first to third transverse tubercles (TST) (Fig. 3). Lateral branch block techniques included 3 needle placements at each individual TST from S1 to S3 using in-plane approaches (Fig. 4). Needles were placed at the level of the periosteum, no more than 1 cm lateral from the lateral border of the PSF by Finlayson et al.^17^ A long-acting local anesthetic (1 ml Marcaine 0.5%, Astra Zeneca) without vasoconstrictors was injected at each needle placement. After the procedure, NRS levels were assessed, and physical and neurological examinations were repeated.

There were 54 patients in whom SIJ pain was confirmed by SIJ blockage, a conservative treatment method, and the electrode conventional radiofrequency denervation method was applied. In those patients with a positive response, defined as temporary pain relief (≥ 50% pain relief at least 3 days after lateral branch block) measured by NRS, the surgeon began conducting the RFN procedure similar to the SIJ block with the radiofrequency electrode (RF cannula needle, length 100 mm or 150 mm with a 10 mm active tip, Cosman Medical, Massachusetts, USA) was placed TST from S1 to S3 under ultrasound. The position of the needle tip was examined by using the Stimulation Program of the Radiofrequency generator machine (Cosman G4, Cosman Medical, Massachusetts, USA). Sensory stimulation was performed at 50 Hz, and the patient was asked to notify the operator in case of tingling, electric shock-like sensation, or tingling. For each grade, paresthesia was achieved ≤ 0.5 V. Then, a motor stimulus was applied at ≤ 2 Hz through the paresthesia points and ensured no muscle contraction in the lower extremities. Afterward, RFN was applied at 80° for 3 min.

**Figure 5:**
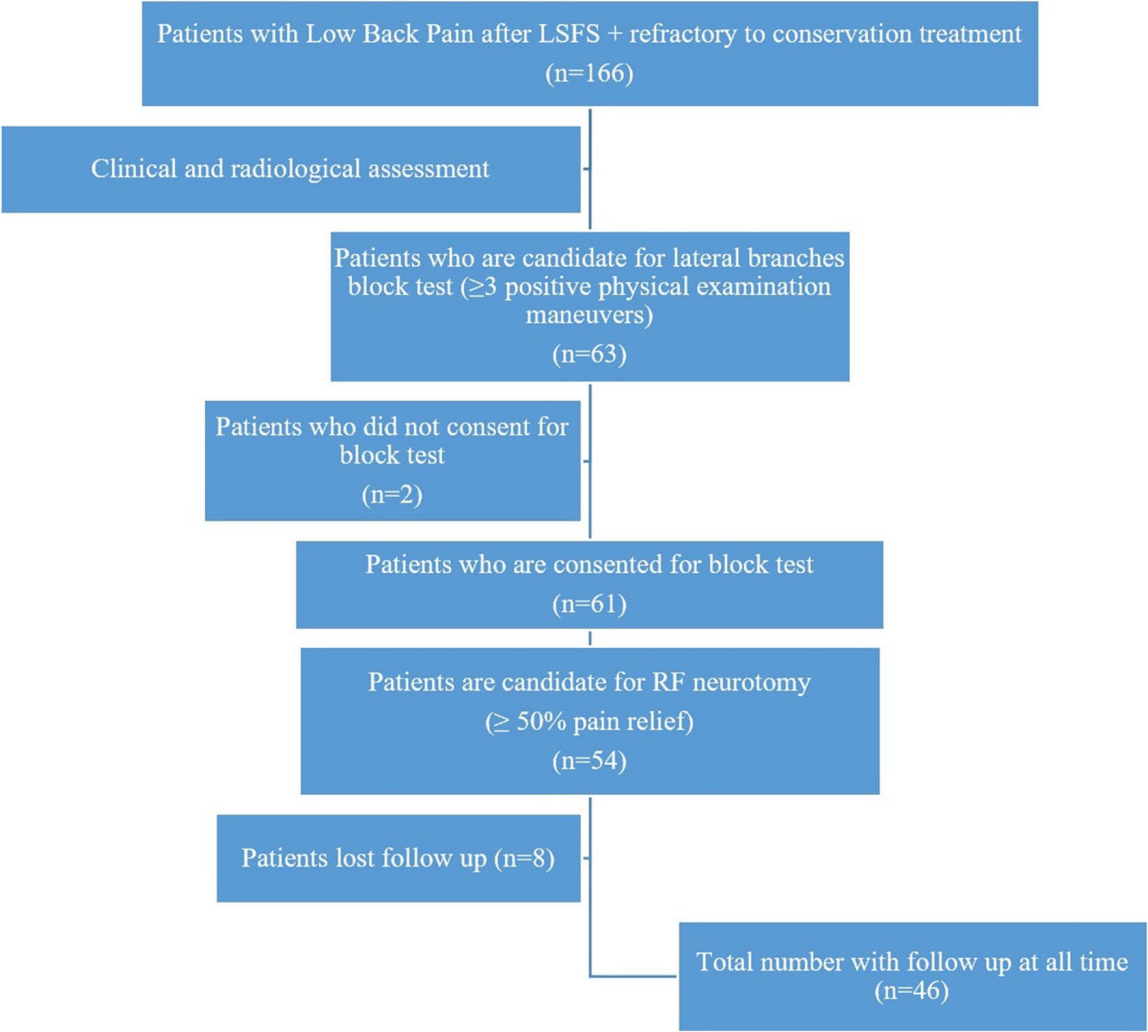
Patient selection flow diagram. (Software: Adobe Inc., 2019. Adobe Photoshop, Available at: https://www.adobe.com/products/photoshop.html).

Vital signs were checked during the procedure. Neurological check-ups of the lower limb included motor examination in form tone, muscle strength, reflexes and sensory examination. Patients were evaluated 2 h after the procedure and discharged with advice to avoid too much bending, lifting heavy weights or walking distances and asked to reexamine at 1 week, 1 month, 6 months and 12 months. The NRS and Oswestry Disability Index (ODI) were recorded at all times. During the period of recruitment (from Jan 2019 to Jan 2022), there were 54 eligible patients. Due to the COVID-19 pandemic and serious effects on Vietnam beginning in May 2021, posterior procedure assessment had to be stopped because of isolation, and 8 patients were lost to follow-up. Therefore, only 46 patients were followed up in our 12-month study, as shown in the flow chart in Fig. 5.

### Measurement of outcome

A physician unaware of the patient's team assignment obtained all outcome data during scheduled follow-up visits. Between the procedure and the initial follow-up, contact between the patient and the investigator should not be allowed except in an emergency.

The patients’ characteristics were sex, age and baseline data upon admission. The data were collected, including the NRS and Oswestry Disability Index (ODI) used by Cohen^3^ and GPE (Global Perceived Effect) by Dutta^18^. The outcome parameter was assessed after one month, 6 months and 12 months using NRS and ODI and after 12 months using GPE.

A 2-point or ≥ 50% reduction on the NRS was deemed to represent clinically meaningful improvements in pain intensity. The Oswestry Disability Index (ODI) calculated 10 items, including pain, individual function and personal comprehensive function. The minimum score for each item is 0 (good state), whereas the highest score is 5 (poor state). The ODI referred to the percentage of the sum of scores from all 10 items out of 50. A positive GPE was defined as an affirmative response to the following three questions:

1. My pain has improved/worsened/stayed the same since my last visit.
2. The treatment I received improved/did not improve my ability to perform daily activities.
3. I am satisfied/not satisfied with the treatment I received and would recommend it to others.

### Ethics considerations

The study was approved by the Institutional Review Board of the University of Medicine and Pharmacy at Ho Chi Minh City and designated 115/ĐHYD-HĐĐĐ. The study was prepared on the ethical principles of the Declaration of Helsinki. The informed consent form will contain extensive information about this trial, including the purpose of the trial, the study design, potential risks and benefits associated with the trial and the participant’s rights and responsibilities.

### Data analysis

Mean, standard deviation, median, interquartile, frequency and percentage were used to describe the data. Chi-squared tests, Fisher’s exact tests and Paired-Sample T-tests were used to evaluate the association between patient characteristics and pain degree. All statistical tests were two-sided with a significance level of 0.05. Continuous data before operation with non-normal distribution were analyzed using the Mann–Whitney U-test, Wilcoxon Signed Rank test. Data were analyzed using the SPSS 25.0 (IBM Corp., Armonk, NY, USA) software.

## Supplementary Information

Supplementary Information.

## Data Availability

All data generated or analysed during this study are included in this published article and its supplementary information file.
